# Export of Extracellular Polysaccharides Modulates Adherence of the Cyanobacterium *Synechocystis*

**DOI:** 10.1371/journal.pone.0074514

**Published:** 2013-09-10

**Authors:** Michael L. Fisher, Rebecca Allen, Yingqin Luo, Roy Curtiss

**Affiliations:** 1 Biodesign Institute, Center for Infectious Diseases and Vaccinology, Arizona State University, Tempe, Arizona, United States of America; 2 Research Informatics Core, Harold & Muriel Block Institute for Clinical & Translational Research at Einstein and Montefiore, Albert Einstein College of Medicine at Yeshiva University, Bronx, New York, United States of America; University of New South Wales, Australia

## Abstract

The field of cyanobacterial biofuel production is advancing rapidly, yet we know little of the basic biology of these organisms outside of their photosynthetic pathways. We aimed to gain a greater understanding of how the cyanobacterium *Synechocystis* PCC 6803 (*Synechocystis*, hereafter) modulates its cell surface. Such understanding will allow for the creation of mutants that autoflocculate in a regulated way, thus avoiding energy intensive centrifugation in the creation of biofuels. We constructed mutant strains lacking genes predicted to function in carbohydrate transport or synthesis. Strains with gene deletions of *slr0977* (predicted to encode a permease component of an ABC transporter), *slr0982* (predicted to encode an ATP binding component of an ABC transporter) and *slr1610* (predicted to encode a methyltransferase) demonstrated flocculent phenotypes and increased adherence to glass. Upon bioinformatic inspection, the gene products of *slr0977, slr0982,* and *slr1610* appear to function in O-antigen (OAg) transport and synthesis. However, the analysis provided here demonstrated no differences between OAg purified from wild-type and mutants. However, exopolysaccharides (EPS) purified from mutants were altered in composition when compared to wild-type. Our data suggest that there are multiple means to modulate the cell surface of *Synechocystis* by disrupting different combinations of ABC transporters and/or glycosyl transferases. Further understanding of these mechanisms may allow for the development of industrially and ecologically useful strains of cyanobacteria. Additionally, these data imply that many cyanobacterial gene products may possess as-yet undiscovered functions, and are meritorious of further study.

## Introduction

The need for alternative and sustainable fuels has become widely accepted, and biofuel production in microalgae and cyanobacteria has been developed to that end. In our laboratory we have engineered the cyanobacteria, *Synechocystis* to produce high levels of fatty acid and to automatically process their own biomass in an inducible manner [1]–[4]. Unlike traditional sources of biofuels, the use of photosynthetic microorganisms does not require the use of cultivatable land or potable water. However, the large culture volumes required to produce biofuels at commercial scale are considerable. Traditional sources of biomass dewatering rely on potentially toxic flocculants, filtration or centrifugation, making biomass dewatering an energy intensive process [5]. In an attempt to diminish the energy requirements for biomass dewatering, cyanobacteria have been constructed with modulated adherence properties with potential for use in biomass harvest and bioremediation [6]–[8].

Both extracellular polysaccharides (EPS) and O-antigen (OAg) are carbohydrate moieties on the cell surface that mediate bacterial protection and provide a mechanism for bacterial cells to interact with their environments [9]–[11]. Between species, OAg is a highly variable, surface-exposed component of lipopolysaccharide in the outer membrane of Gram-negative bacteria. This key structure modulates susceptibility to phage [12], [13], surface charge [14] and permeability of the outer membrane [14]. Similarly, EPS is a large, polymeric carbohydrate structure that serves to protect cells from environmental stress such as UV irradiation [15], heavy-metal toxicity [8], [16], [17], osmotic stress [8], [18] and desiccation [19].

In this study, we completed a whole genome comparison of *in silico* translated genes from *Synechocystis, E. coli* K-12 and *S.* Typhimurium LT2. We searched for genes in *Synechocystis* that showed homology to genes involved in modulating the cell surface moieties in other species. Two of the genes we identified were *slr0977* and *slr0982*. These genes encode products that are homologous to the Wzm and Wzt proteins in *E. coli.* Together, these gene products comprise the permease and oligosaccharide binding proteins that function together as a ABC-transporter [20], [21].

We noted that *slr0977* and *slr0982* were located in a cluster of contiguous genes that were annotated as OAg transport genes in the Cyanobase genome database [22], [23]. Subsequent mutation of these genes resulted in flocculating strains with modulated adherence properties. Surprisingly, neither the OAg structure nor its composition was altered in these mutants. Rather, we provide evidence that the phenotypes exhibited by these mutants are due to altered extracellular polysaccharide. Together with previously published studies, these data indicate multiple mechanisms for autoflocculation through disruption of EPS biosynthesis and/or export. This understanding is important for the construction of autoflocculating mutant strains.

## Materials and Methods

### Bioinformatics Searches and Analyses

The complete genomes for *E. coli* K-12 (NC_000913.2), *S.* Typhimurium LT2 (NP_459707.1), and *Synechocystis* 6803 (BA000022.2) were downloaded from NCBI GenBank and gene information was extracted using Perl scripts. The COG database [24] was also used in categorizing genes. All of the features were stored in a MySQL database. For gene identification, we used BLASTP implemented in NCBI blast-2.2.18 to identify possible homologous genes of *E. coli* or *Salmonella* in the *Synechocystis* genome with a threshold e-value less than 1.0^–4^. Three methods were used to define genes of synthesis and assembly of cell wall components: (1) Based on a set of genes in *E. coli* K-12 and *S.* Typhimurium LT2, the homologous genes in *Synechocystis* using were determined. (2) a set of genes based on the functional descriptions of the COG database were determined, and then the homologs in *Synechocystis* were identified using BLASTP (3) We searched for genes directly based on the functional annotation of *Synechocystis*. Thus, 519 gene products potentially involved in the synthesis and assembly of the *Synechocystis* cell wall and cell-surface macromolecular components were identified.

### Bacterial Growth Conditions

Cultures were inoculated to a starting OD_730_ of 0.1 in BG-11 medium [25] from starter cultures. Cultures were incubated at 30°C with 40 µmol photons m^−1^ s^−1^. Kanamycin was added at a concentration of 50 µg/mL when appropriate. To assess the effect of NaCl on EPS mutants, *Synechocystis* cultures were inoculated in triplicate to an OD_730_ 2 mL of BG-11 or BG-11+0.8M NaCl in test tubes. Every 24 h cultures were gently agitated with a vortex until adherent cells were fully in suspension. The OD_730_ was then measured and recorded.

### Vector Construction

DNA manipulation was carried out using standard procedures [26]. Suicide vectors were constructed in a two-step process. For each gene deletion, PCR primers (Table 1) were used to amplify ∼400bp of genomic DNA from the flanking region of of each gene. Flanking regions were stitched together by a modified overlap extension PCR as previously described [27]. Briefly, BamHI and NdeI restriction sites were generated between the two flanking sequences. Thus, we created the plasmids pψ508 (*slr0977* flanking regions), pψ512 (*slr1610* flanking regions), ψ514 (*sll0574-5* flanking regions) and pψ560 (*slr0982* flanking regions). The Kan^R^-SacB cassette from pPsbA2ks [28] was purified following digestion with BamHI and NdeI and ligated into pψ508, pψ512, ψ514 and pψ560 to generate pψ509 (Δ*slr0977*) pψ513 (Δ*slr1610*), pψ515 (Δ(*slr0977;*Δ*sll0574-5*)) and pψ561 (Δ*slr0982*) respectively. In the case of pψ560, an NdeI site native to the 3′ flanking region was removed by introducing a silent mutation using QuikChange II Site-Directed Mutagenesis Kit (Agilent Technologies) according to the manufacture’s protocol, using primers MLF-68 and MLF-69.

**Table 1:** Primers used in this study.

Primer Name	Primer sequence	Description	Plasmids constructed
MLF-1	tttatgccactaggttcc	5′ primer for upstream region of *slr0977*	pψ508 and pψ618
MLF-2	ggatcctttaaaccccatatgcatacttgaggtcaatttttg	3′ primer for upstream region of *slr0977*	
MLF-3	catatggggtttaaaggatcctaaccatggcaacaaac	5′ primer for downstream region of *slr0977*	
MLF-4	ccttcctcaactcttcgttg	3′ primer for downstream region of *slr0977*	
MLF-9	ctactatgggaagatttttg	5′ primer for upstream region of *slr1610*	pψ511 and pψ620
MLF-10	ggatcctttaaaccccatatgcactcaatccctaggcgag	3′ primer for upstream region of *slr1610*	
MLF-11	catatggggtttaaaggatcctgttagaatgttgagcagg	5′ primer for downstream region of *slr1610*	
MLF-12	tcaagaatttgacccag	3′ primer for downstream region of *slr1610*	
MLF-13	agtcaactcggaattgt	5′ primer for upstream region of *slr0982*	pψ560 and pψ619
MLF-14	ggatcctttaaaccccatatgcgaatgactgtatcagacat	3′ primer for upstream region of *slr0982*	
MLF-15	catatggggtttaaaggatccattgcatgaaagctgtaattc	5′ primer for downstream region of *slr0982*	
MLF-16	attagaccgccatcaccg	3′ primer for downstream region of *slr0982*	
MFL-68	caattattttctacacatgtccgatgtaacc	5′ NdeI SDM removal in flanking region 2 of the*slr0982* construct	
MFL-69	tgttacatcggacatgtgtagaaaataattg	3′ NdeI SDM removal in flanking region 2 of the*slr0982* construct	
MLF-17	ggtttgaacagaatcaag	5′ primer for upstream region of *sll0574-5*	pψ513
MLF-18	ggatcctttaaaccccatatgcggtagcgaaagagccat	3′ primer for upstream region of *sll0574-5*	
MLF-19	catatggggtttaaaggatccccccaataattctggcaag	5′ primer for downstream region of *sll0574-5*	
MLF-20	ccaccttagttactccatag	3′ primer for downstream region of *sll0574-5*	

To rule out second-site mutations that might have accumulated during the construction of mutant strains, we constructed vectors for genomic complementation. Complementation was carried out by replacing the Kan^R^-SacB in each mutant with the wild-type allele. Complementation vectors pψ618, pψ619 and pψ620 were constructed to complement the mutations Δ*slr0977*, Δ*slr0982*, Δ*slr1610* in SD506, SD553 and SD507, respectively. To construct these vectors, DNA from SD100 was amplified with primer pairs MLF-1/MLF-4, MLF-5/MLF-8 and MLF-9/MLF-12. The PCR products were ligated into pJET1.2 to generate pψ618, pψ619 and pψ620, respectively. SD553 was transformed with pψ619 to generate SD563. SD507 was transformed with pψ620 to generate SD564. Several attempts were made to complement the Δ*slr0977* strain by transformation of SD506 with pψ618. However, no transformants were recovered.

### Transformation and Complementation

Mutants of *Synechocystis* were generated as previously described [1]. Briefly, 4 µg of suicide vector was added to 100 µL of a late log phase culture of and incubated overnight in BG-11 without antibiotic. The entire transformation mixture was then spread onto a Nuclepore Track-etch membrane (Whatman #111707) on a BG-11 plate. These were then incubated for three days at 30°C with 40 µmol photons m^−1^ s^−1^. Following this incubation, membranes were transferred to a BG-11 plate containing 100 µg/mL kanamycin and incubated until single colonies appeared. To ensure complete segregation of the mutants, colonies were patched onto BG-11+6% sucrose and BG-11+100 µg/mL kanamycin. Kanamycin resistant colonies that were sucrose sensitive were further verified by PCR to ensure complete segregation.

To complement mutants in SD553 and SD507, 100 µL late log cultures of each strain were transformed with 4 µg of either pΨ619 or pΨ620, respectively to generate SD563 and SD564. Following an overnight incubation at 30°C with 40 µmol photons m^−1^ s^−1^, transformation mixtures were resuspended in 1mL of BG-11 and allowed to recover for three days. Following the recover step, 100 µL of culture was then plated on BG-11+6% sucrose and incubated until single colonies arose. Isolated colonies were then patched onto BG-11+6% sucrose and BG-11+100 µg/mL kanamycin. Colonies that were sucrose resistant and kanamycin sensitive were verified by PCR and phenotypic assays (see crystal violet adherence assay below) for complete segregation.

### Crystal Violet Adherence Assay

*Synechocystis* cultures were inoculated at an OD_730_ of 0.1 in 2 mL of BG-11 medium in test tubes. Kanamycin was added at a concentration of 50 µg/mL when appropriate. In each experimental replicate, static cultures were grown in triplicate for 96 hours at 30°C with 40 µmol photons m^−1^ s^−1^. The adherence of three cultures was then measured as follows: The culture medium containing non-adherent cells was decanted from each of the tubes. Each tube was gently washed 2× with 2 mL of BG-11. Tubes were then stained with 1% crystal violet in _dd_H_2_O for 15 min. The tubes were then gently rinsed 3× with _dd_H_2_O. Adherent cells were resuspended in 1 mL of DMSO and vortexed vigorously. The OD_630_ of each tube was then measured. Each strain was analyzed in triplicate. Representative data of three separate experiments are presented.

### LPS Extraction and Analysis

LPS extraction was carried out as described [29]. Briefly, 25 mL cultures of *Synechocystis* were inoculated to an initial cell density of 0.1 OD_730_ in 50 mL of BG-11 in 125 mL Erlenmeyer flasks for 4 days at 30°C with 40 µmol photons m^−1^ s^−1^, shaking at 115 rpm. Cultures were normalized for cell density and centrifuged at 6000 rpm in a Sorvall SS-34 rotor for 20 min. Bacterial pellets were resuspended in 5 mL TRI reagent (Sigma) in 15 mL conical tubes, vortexed and incubated at room temperature overnight with gentle shaking. Then, 1 mL of chloroform was added to each tube which was then vortexed well and incubated at room temperature for 15 min. Samples were then centrifuged at 11,000 rpm in a Sorvall SS-34 rotor for 10 min. The aqueous phase was transferred to a fresh tube and the organic phase was back-extracted with 2 mL of _dd_H_2_O. Samples were frozen and lyophilized overnight. Samples were then resuspended in cold 0.375 M NaCl in 95% ethanol, transferred to 1.5 mL centrifuge tubes and washed 2× in 0.375 M NaCl in 95% ethanol, resuspended in 1mL of cold 100% ethanol and lyophilized overnight. Samples were then washed three times in cold Folch reagent (2∶1 CHCl_3_:MeOH), lyophilized and resuspended in 100 µL _dd_H_2_O. LPS was analyzed by SDS-PAGE on a 4–15% gradient gel (Biorad) and then visualized by silver staining [30].

### EPS Extraction and Analysis

EPS was extracted by mechanical disruption as described [31]. Cells were grown to early stationary phase (OD_730_ ∼1.0) and normalized for equal OD_730_. Fifty milliliters of cells were centrifuged for 20 minat 10,000×g (Sorvall SS-34). Samples were resuspended in 10 mL BG-11 and fixed with 60 µL of formaldehyde (Sigma) overnight at 4°C. 4 mL of 1 M NaOH was added to each sample, which was then vigorously agitated with a vortex for 15 sec. Samples were centrifuged for 20 min at 20,000×g (Sorvall SS-34). The supernatants were frozen, lyophilized and resuspended in 2 mL of water, each. At this point, samples were dialyzed three times against 4 L of water, lyophilized overnight and resuspended in 100 µL of water. 10 µL of each sample was loaded onto a 12% Mini-PROTEAN® TGX™ Precast Gel (BioRad #456-1045) and stained with Alcian Blue. Briefly, gels were fixed with 12.5% trichloroacetic acid for 30 min and rinsed with water. Following incubation in a solution containing 1% periodate and 3% acetic acid, gels were washed five times in water. Gels were then incubated in 0.5% potassium disulfite for thirty min and washed three times in water. At this point, gels were stained with a solution of 0.5% Alcian blue in 3% acetic acid overnight. Prior to visualization, gels were destained with 7% acetic for 5 min or until EPS bands were visible.

### Outer Membrane Protein Analysis

Outer membrane proteins were extracted as described [32]. Cultures were grown under static conditions as described above. Fifty milliliters of culture at OD_730_ 0.4 were centrifuged and the outer membrane faction was purified. Membrane fractions were resuspended in 20 µL of Laemmli buffer with 5.5% β-mercaptoethanol (Sigma). Each sample was loaded in its entirety onto a 12% SDS-PAGE gel and stained with Coomassie brilliant blue.

### Glycosyl Composition Analysis of OAg and EPS

Glycosyl composition analysis was performed by combined gas chromatography/mass spectrometry (GC/MS) of the per-*O*-trimethylsilyl (TMS) derivatives of the monosaccharide methyl glycosides produced from the sample by acidic methanolysis. Between 200 and 300 µg of each sample was used for the analysis. The samples were placed into test tubes and 20 µg of inositol was added. Methyl glycosides were then prepared from the dry samples by methanolysis in 1 M HCl in methanol at 80°C (17 hours), followed by re-*N-*acetylation with pyridine and acetic anhydride in methanol (for detection of amino sugars). The samples were then per-*O*-trimethylsilylated by treatment with Tri-Sil (Pierce) at 80°C (0.5 hours). These procedures were carried out as previously described [33], [34]. GC/MS analysis of the TMS methyl glycosides was performed on an Agilent 7890A GC interfaced to a 5975C MSD, using a Agilent DB-1 fused silica capillary column (30 mm × 0.25 mm ID).

## Results and Discussion

We wished to ascertain what genes in *Synechocystis* were likely involved in the biosynthesis of structural components of cell surface structures. To this end, whole genome comparisons of *Synechocystis* were carried out against *E. coli* K-12 and *S. enterica* serovar Typhimurium LT2. We identified over 500 putative genes potentially involved in the synthesis and assembly of the *Synechocystis* 6803 cell wall and cell-surface macromolecular components. Additionally, we identified gene products involved in LPS biosynthesis, polysaccharide biosynthesis, extracellular appendages, cell wall biosynthesis, metabolite biosynthesis and peptidoglycan biosynthesis (Tables S1).

The nomenclature for OAg biosynthesis has changed over the years [35]. Whereas the OAg ABC transporter components were once designated *rfbA* (permease component) and *rfbB* (ATP binding component), some of these are now given designations *wzm* (permease component) and *wzt* (ATP binding component) [35]. Our analysis revealed that *Synechocystis* contains two systems similar to the *wzm/wzt* genes that encode OAg transporters in *E. coli*. Genes encoding products annotated as involved in OAg synthesis and assembly, are present in the same gene cluster. Specifically, *slr0983*, *slr0984* and *slr0985* were also assigned the *“rfb”* designations *rfbF, rfbG* and *rfbC*, respectively. Interestingly, a similar gene arrangement (*rfbFGC)* was bioinformatically identified in *Azotobacter vineladnii* [36]. Following *slr0985* in the *Synechocystis* gene cluster described here is *slr1610,* which is annotated as encoding a methyltransferase (Table 2).

**Table 2:** Bioinformatic analysis of the *slr0977 gene cluster.*

Synechocystis gene	Cyanobase Annotation	Cyanobase function	E. coli Gene Name	E. coli Gene Function	Protein Identity^c^
slr0977	rfbA	ABC transporter, permease component	wzm	ABC transporter membraneprotein	71/259 (27%)
slr0978-81	hypothetical	unknown	–	–	–
slr0980	hypothetical	unknown	–	–	–
slr0981	hypothetical	unknown	–	–	–
slr0982	rfbB	polysaccharide ABC transporterATP binding subunit	wzt	ATP binding component ofABC-transporter	100/232 (43%)
slr0983	rfbF	glucose-1-phosphate cytidylyltransferase	NA^a^	dTDP-glucose 4,6-dehydratase 2	89/358 (25%)
slr0984	rfbG	CDP-glucose 4,6-dehydratase	NA	dTDP-4-deoxyrhamnose-3,5-epimerase	97/341 (28%)
slr0985	rfbC	dTDP-4-dehydrorhamnose 3,5-epimerase	rmlC	dTDP-4-dehydrorhamnose 3,5-epimerase	100/165 (60%)
slr1610	hypothetical	putative C-3 methyltransferase	NA	bifunctional 3-demethylubiquinone-9 3-methyltransferase	32/142 (23%)
sll0574	rfbA	permease protein of lipopolysaccharideABC transporter	wzm	ABC transporter membrane protein	72/266 (27%)
sll0575	rfbB	lipopolysaccharide ABC transporter ATP binding subunit	wzt	ATP binding component of ABC-transporter	86/239 (36%)

a. Not Assigned.

The genes *slr0977* and *sll0574* encode homologs of the integral membrane protein Wxm. The *slr0977* and *sll0574* gene products possessed 24% and 29% identity to Wzm in *E. coli*, respectively (Table 1). Similarly, *slr0982* and *sll0575* are annotated as *rfbB* [22]. These gene products are 34% and 41% identical to the ABC protein, Wzt in *E. coli*. Wzt possess a C-terminal nucleotide binding cassette and an N-terminal carbohydrate binding domain [37]. While the C-terminal, nucleotide binding regions of the *slr0982* and *sll0575* gene products are highly homologous, there is little homology at the N-terminal region of these predicted gene products. This lack of N-terminal homology is likely indicative of differential substrate specificity for the type of polysaccharides transported by these systems.

### Disruption ABC Transporters Leads to Flocculent Cultures with Modulated Adherence Properties

**Figure 1:**
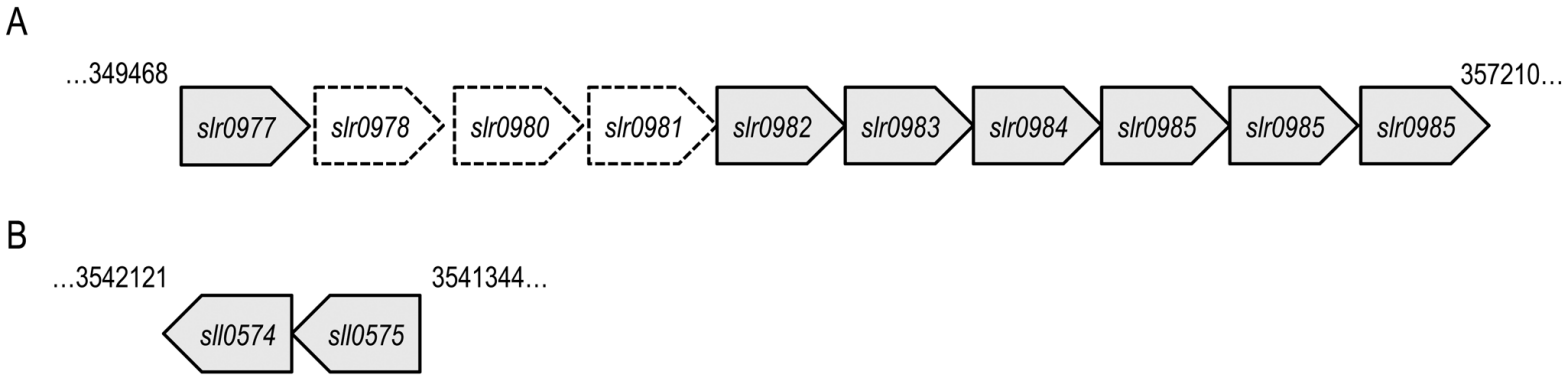
Genetic organization of the *slr0977* gene cluster. (A) *slr0977* and *slr0982* are predicted to encode a permease and ATPase components of an ATP-transporter, respectively. *slr0978-0981* encode hypothetical proteins of unknown function. *slr1610* is a predicted methyltransferase. (B) *sll0574* and *sll0575* are predicted to encode a permease and ATPase components of an ATP-transporter, respectively.

In *Synechocystis,* genes encoding products that function in the same biosynthetic pathway are not consistently organized in operons [22]. It is interesting therefore, that the genes *slr0977* and *slr0982* are part of a nine-gene cluster in *Synechocystis* (Fig. 1A). These two ABC transporter components are separated by *slr0978*, *slr0980*, and *slr0981,* which are hypothetical genes with no known function. These genes are followed by *slr0983* (glucose-1-phosphate cytidylyltransferase), *slr0984* (CDP-glucose 4,6-dehydratase), *slr0985* (dTDP-4-dehydrorhamnose 3,5-epimerase) and *slr1610* (putative C-3 methyltransferase). We also observed that the genes *sll0574* and *sll0575* located distantly on the complementary strand of the genome (Fig. 1B) encoded for homologs of Wzm and Wzt respectively.

**Figure 2:**
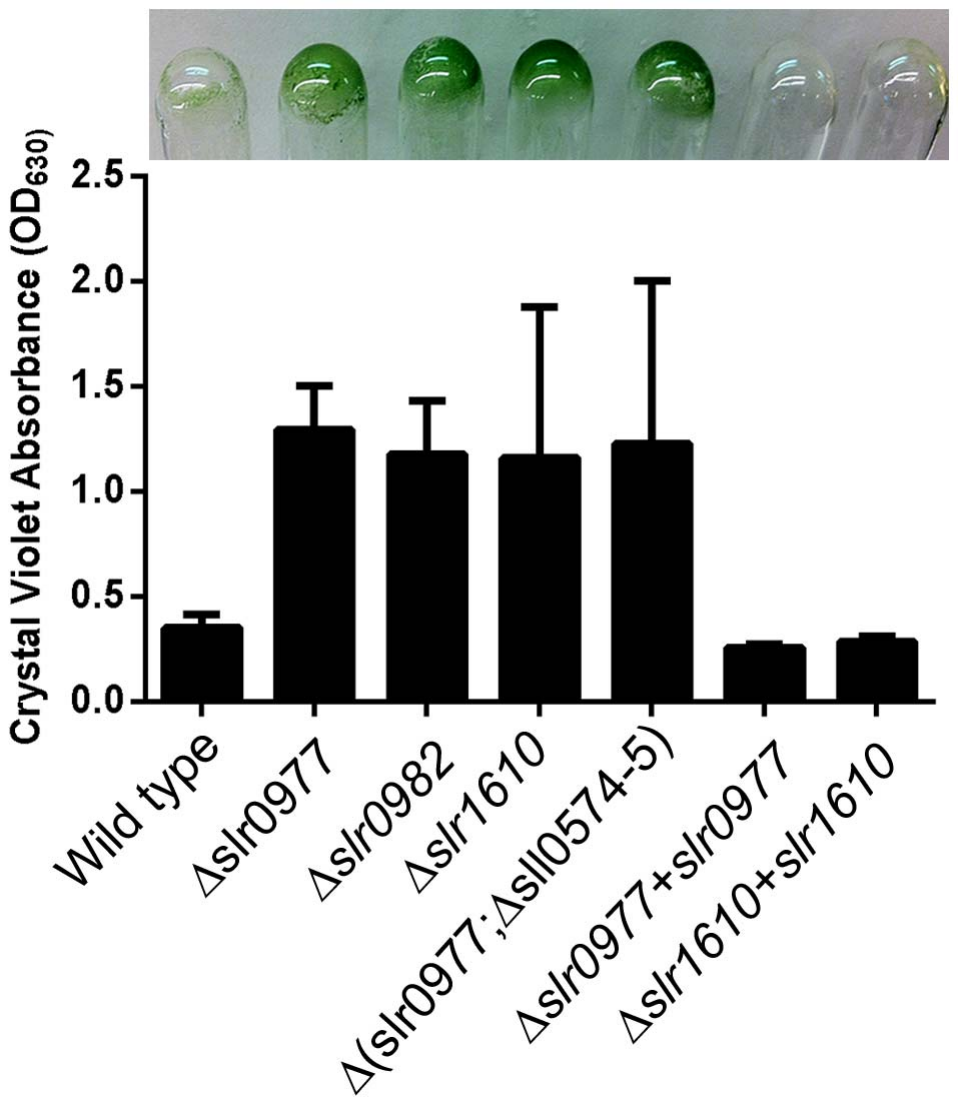
Adherence of *Synechocysis* mutants to glass culture vessels. Quantification was carried out by spectrophotometry of crystal violet staining of attached cells. Strains were grown statically in 2-11 at 30°C with 40 µmol photons m^−1^ s^−1^ for 4 days. Data are the combination of at least three biological replicates. Representative data of strains adhering to glass culture vessels (inset). Error bars indicate the standard deviation from the mean.

To investigate the role of *slr0977* and *slr0982* in cell surface modification, we generated mutants by deleting these genes in the SD100 background [1]. Mutant strains lacking different combinations of these genes were created through allelic replacement with a kanamycin-resistance cassette and the counter-selectable *sacB* gene (Table 3) [38]. The growth rates of Δ*slr0977*, Δ*slr0982*, Δ*slr1610*, or the triple mutant Δ(*slr0977*;Δ*sll0574-5*) were identical to wild type. However, mutants demonstrated readily apparent flocculent phenotypes and adherence to glass culture vessels (Fig. 2).

**Table 3:** Strains and Plasmids.

Strain	Relevant Genotype	Source
SD100	*Synechocystis* PCC 6803	[1]
SD506	Δ*slr0977*	this study
SD553	Δ*slr0982*	this study
SD507	Δ*slr1610*	this study
SD565	Δ(*slr0977*;Δ*sll0574-5*)	this study
SD563	Δ*slr0982+ slr0982*	this study
SD564	Δ*slr1610*+ *slr1610*	this study
pJet1.2	general cloning vector (pUC19 derivative)	Fermentas
pPbsA2ks	Source of kanamycin-resistance-sacB cassette	[41]
pψ508	suicide vector for counterselecting sacB in SD506	this study
pψ509	suicide vector for constructing SD506	this study
pψ510	suicide vector for counterselecting sacB in SD501	this study
pψ511	suicide vector for constructing SD501	this study
pψ512	suicide vector for counterselecting sacB in SD507	this study
pψ513	suicide vector for constructing SD507	this study
pψ514	suicide vector for counterselecting sacB in SD565	this study
pψ515	suicide vector for constructing SD565	this study
pψ560	suicide vector for counterselecting sacB in SD553	this study
pψ561	suicide vector for constructing SD553	this study
pψ618	suicide vector for complementing slr0977mutation in SD506	this study
pψ619	suicide vector for complementing slr0982mutation in SD507	this study
pψ620	suicide vector for complementing slr1610mutation in SD507	this study

We quantified the adherence of each mutant to the growth vessel by the crystal violet binding assay (Fig. 2). All mutant strains tested were significantly better able to adhere to glass culture vessels. Following four days of static growth in a two milliliter culture, the mutant strains Δ*slr0977*, Δ*slr0982*, and Δ*slr1610* all demonstrated a 4-6 fold increase in binding (Fig. 2). The adherence of these strains was specific to glass vessels. Mutant strains grown in polystyrene flat-bottom 96 well plates (Costar or Nunc MaxiSorp®) did not demonstrate increased adherence relative to wild-type (data not shown). The ability of these mutants to flocculate and not adhere to plastic may have industrial applications. This is particularly true of production in closed photobioreactors (PBR), which are often constructed from plastics. Such flocculent strains would decrease the energy requirements for centrifugation or filtration during biofuel production. Concomitantly, such phenotypes may eliminate the risk of biofouling by cyanobacterial growth on the PBR surface.

It is intuitive that Δ*slr0977 and* Δ*slr0982* would demonstrate similar phenotypes given that they are both lacking genes encoding products in the same transport system. The failure to transport the same substrate likely accounts for this phenotype. It is interesting that the Δ*slr1610* mutant demonstrated a nearly identical phenotype to both the Δ*slr0977 and* Δ*slr0982* strains. The *slr1610* gene encodes a putative methyltransferase. In OAg production, a methyltransferase is responsible for terminating carbohydrate polymerization [22]. Additionally, methylation of the carbohydrate chain is important for recognition by the ABC transporter for subsequent transport [39]. Given this, one parsimonious model is that in the absence of the *slr1610* gene product, larger carbohydrate moieties are synthesized by the mutant than in wild-type. It is likely that such long carbohydrate chains would be unable to properly interact with the *slr0977/slr0982* gene products for extracellular transport. It is unlikely that the phenotypes of these mutants are due to polar effects created while constructing the *slr0977* or *slr0982* deletions. This is evidenced by the fact that a similar mutation Δ(slr0978-81) (located between the *slr0977* and slr*0982* ORFs) was indistinguishable from wild-type in adherence, flocculence and growth (data not shown).

### Mutant Adherence Occurs as a Monolayer

**Figure 3:**
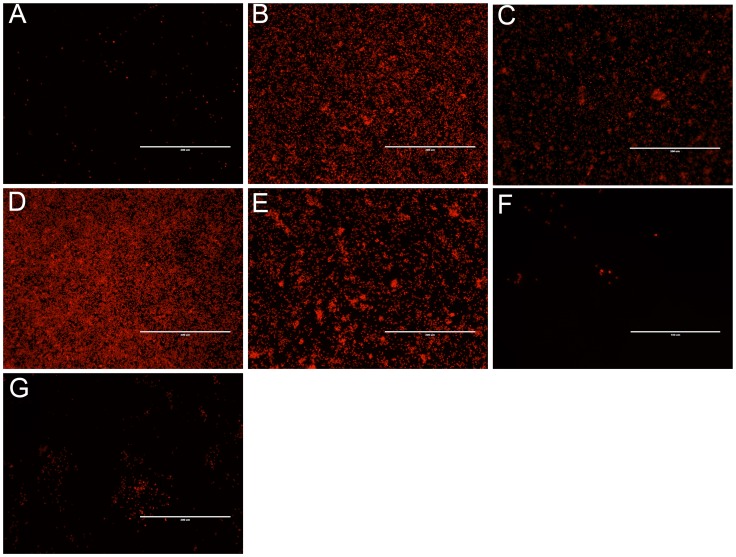
Adherence of mutant strains to glass coverslips after 4 days of growth. Following four days of static growth on sterile glass coverslips, slides were washed gently with water and *Synechocystis* autofluorescence was visualized by fluorescence microscopy. (A) wild-type, (B) Δ*slr09777*, (C) Δ*slr0982,* (D) Δ*slr1610* (E) Δ(*slr0977;* Δ*sll0974-5)*, (F) Δ*slr0982+ slr0982*, (G) Δ*slr1610+slr1610*. White scale bars indicate 200 µm.

To further examine the adherence of cell to glass, wild-type and mutants were grown on sterile coverslips for four days in static culture and examined by fluorescence microscopy (Fig. 3). Consistent with crystal violet staining, wild-type cells adhered poorly to glass (Fig. 3A), whereas Δ*slr0977,* Δ*slr0982*, Δ*slr1610* and Δ(*slr0977*;Δ*sll0574-5*) readily adhered to coverslips (Fig. 3B-E, respectively). Furthermore, the adherence phenotypes of the Δ*slr0982* or Δ*slr1610* mutants is unlikely due to compensatory mutations as strains complemented with a wild-type allele were indistinguishable from wild-type (Fig. 2 and Fig. 3E-F).

We did not observe complex, three-dimensional architecture consistent with biofilm formation [40]. Rather, we saw monolayers of cells strongly adhered to glass (Fig. 3A). These data are consistent with the idea that disruption of these gene products leads to a change in the charge of the cell surface. In fact, EPS biosynthesis mutants lacking *sll1581* (*gumB)* and *sll5052* (*gumC*) aggregated out of solution and had altered cell surface charges [8].

### OAg Structure and Composition is Unaffected by Mutations in Δ*slr0977*, Δ*slr0982*, Δ*s*l*r1610* and Δ(*slr0977*;Δ*sll0574-5*)

**Figure 4:**
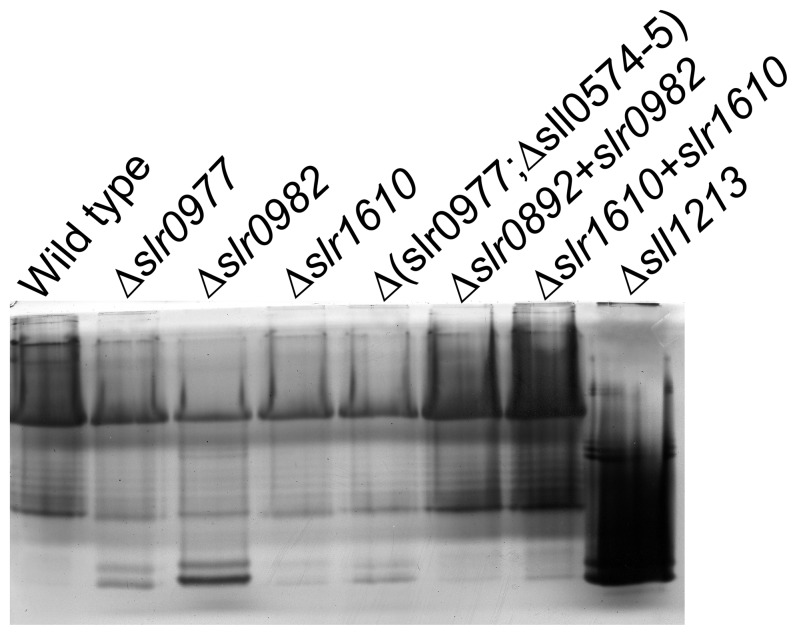
Purified LPS analysis from the adhesive mutants: Δ*slr0977,* Δ*slr0982,* Δ*s*l*r1610* and Δ(*slr0977;*Δ*sll0574-5*). LPS was purified from wild-type (WT) or the corresponding mutants. Samples were analyzed by 12% SDS-PAGE and silver staining.

To determine if OAg was responsible for the modulated adherence and flocculation of the mutants generated, total LPS was extracted from mid-log cultures of wild-type or mutant strains and analyzed by SDS-PAGE (Fig. 4). A previously characterized fucose synthase mutant (Δ*sll1213*) [41] was also processed as a positive control for disrupted OAg structures. As expected, the fucose synthase mutant demonstrated marked differences in size and intensities of purified LPS species from the wild-type strain. However, LPS from the mutant strains demonstrate very little difference from wild-type (Fig. 4). Occasionally, some variation in band intensities was observed between the mutant strains and wild-type (e.g. Fig. 4 low molecular weight bands). However, these differences were sporadic and likely due to minor variations in growth stage from one experiment to another, rather than the introduced mutations.

Although there were no obvious differences in size of the OAg derived from Δ*slr0977*, Δ*slr0982*, Δ*slr1610* or Δ(*slr0977*;Δ*sll0574-5*), it seemed possible that compositional changes in OAg accounted for the phenotypes of these strains. Therefore, purified OAg was analyzed by gas chromatography to determine its composition (Table 4). The results of the overall composition of *Synechocystis* OAg are consistent with similar analyses [42]. Glucose was the major component in all wild-type and mutant strains (62.1–76.5%). OAg from mutant and wild-type strains contained similar amounts of glucose as well as of xylose, fucose, galactose, and mannose (Table 4).

**Table 4:** Gas chromatographic analysis of OAg components^a^.

	WT		*Δslr0977*		*Δslr0982*		*Δslr1610*	
Sugar	Mol %	SD	Mol %	SD	Mol %	SD	Mol %	SD
glucose	66.2	8.2	62.1	0	54.1	17	76.5	5.7
xylose	10	0.5	19.4	14.8	13.3	4	8.7	1.2
fucose	9.9	4	5.4	7.6	7.7	8.6	7.8	5.4
galactose	7.3	8.3	1.4	2	6.2	5.1	4.7	4.9
mannose	3.2	4.5	9.4	1.8	3.2	4.5	2.4	3.4
ribose	2.1	1.2	1	1.3	0		0	0
rhamnose	1.5	2.1	0.7	0.9	15.6	22	0	0
N-Acetyl Galactosamine	0	–	0.3	0.4	0	–	0	–
N-Acetyl Glucosamine	0	–	0.5	0.7	0	–	0	–
N-Acetyl Mannosamine	0	–	0	–	0	–	0	–
glucuronic Acid	0	–	0	–	0	–	0	–
galacturonic acid	0	–	0	–	0	–	0	–
arabinose	0	–	0	–	0	–	0	–

a Composition of OAg from the indicated strain. Data are presented as the Mol% of each sugar from the total carbohydrate extracted. Sums may not add to 100% due to rounding.

Although no significant differences were observed in the OAg profile of the Δ*slr0982* mutant, increased concentrations of sugars such as rhamnose were detected. These differences were sporadic between experiments, but it is interesting to speculate that the *slr0977* gene product may be interacting non-specifically with unidentified homologs of the *slr0982* gene product (*i.e.* other ABC transporter components). Since such *slr0982* homologs would have affinity for different carbohydrates, it may explain the sporadic display of unusual sugars in the OAg of this mutant. We have identified 157 putative glycosyl transferases in *Synechocystis* with homology to gene products of *E. coli* and *Salmonella* (Table S1). It is unclear how many of these function in EPS biosynthesis. By engineering strains that have altered glycosyl transferase profiles, it may be possible to engineer altered EPS for designer purposes such as re-capturing waste products from waste water [8], [16], [17].

### EPS is Altered in Strains Δ*slr0977,* Δslr0982, Δ*slr1610* and Δ(*slr0977*; *sll0574-5*)

**Figure 5:**
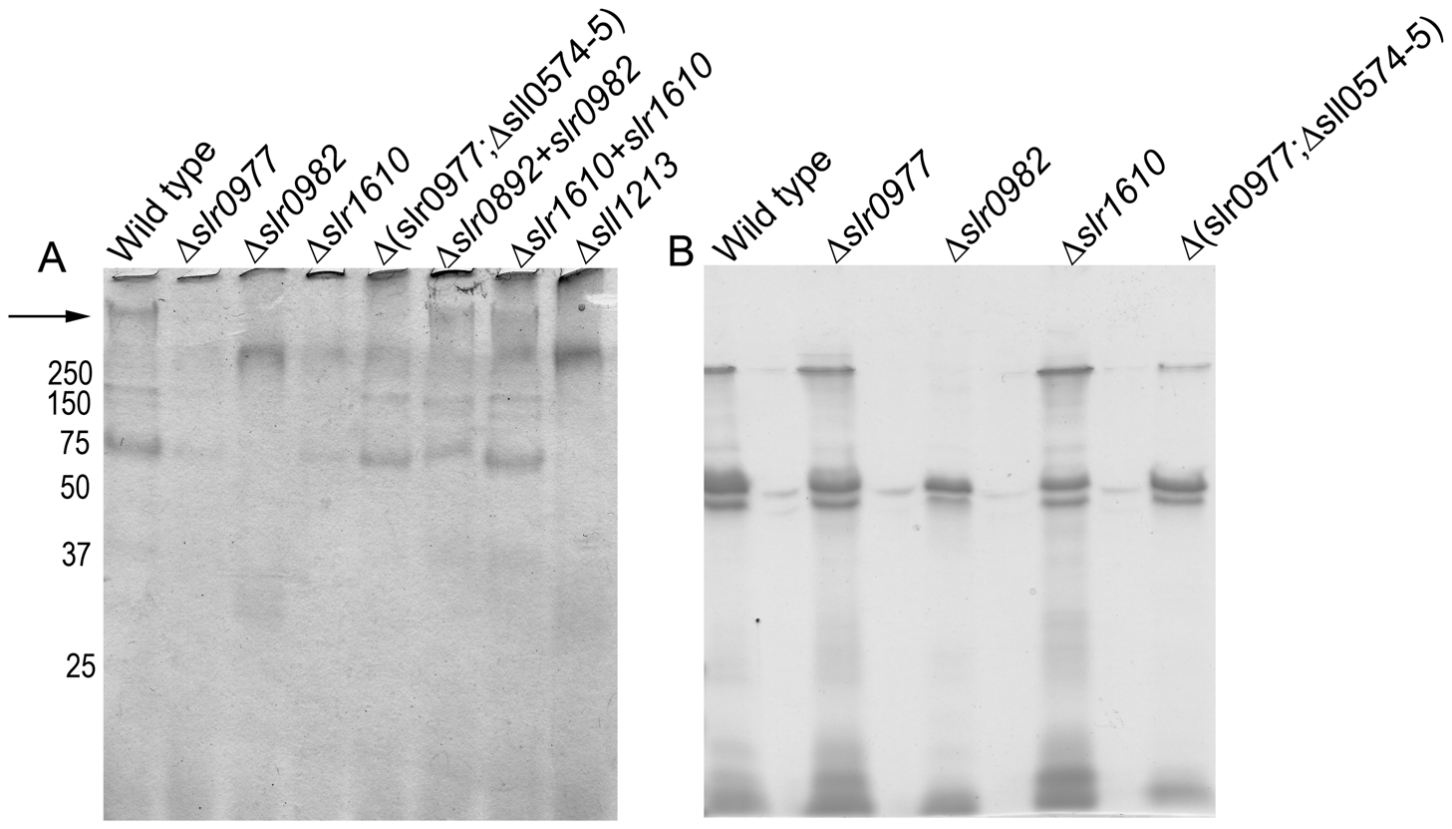
EPS and OMP profiles are effected by mutations in Δslr0977, Δslr0982, Δslr1610 or in a triple mutant Δ(slr0977;Δsll0574-5). (A) EPS as purified from wild-type (WT) or the corresponding mutants by mechanical extraction. EPS from a Δslr1213 strain is known to be deficient in EPS production and was loaded as a control. Samples were analyzed by SDS-PAGE and Alcian Blue staining. Black arrow indicates major EPS band. (B) OMP profile of wild-type or mutant Synechocystis as analyzed by SDS-PAGE and Coomassie brilliant blue staining. Molecular weights of protein standards are indicated for both analyses are indicated by numbers on the left.

Surprisingly, OAg structure was not disrupted in the mutant strains Δ*slr0977,* Δslr0982, Δ*slr1610* and Δ(*slr0977*;Δ*sll0574-5*). It seemed likely, therefore, that these gene products play a role in the export of other carbohydrate substrates. To determine if these gene products functioned in EPS biosynthesis, EPS was extracted from stationary phase cultures of wild-type, Δ*slr0977,* Δ*slr0982*, Δ*slr1610* and Δ(*slr0977*;Δ*sll0574-5*) strains. Samples from each culture were analyzed by SDS-PAGE and Alcian blue staining. The Δ*sll1213* mutant had been previously characterized as an EPS mutant [41] and was used as a positive control for EPS disruption (Fig. 5). The major band from EPS extraction (See Fig. 5, black arrow) is a large molecular weight polymer that runs above 250 kDa. The mutant strains Δ*slr0977,* Δ*slr0982*, Δ*slr1610* and Δ(*slr0977*;Δ*sll0574-5*) all lacked the major EPS band. However, EPS from all mutants tested displayed a smaller EPS band similar to that from the Δ*sll1213* strain, previously demonstrated to produce altered EPS [41].

Given that the mutants we analyzed were deficient in EPS export compared to wild-type, we determined the composition of mutant EPS. Consistent with altered EPS sizes detected in SDS-PAGE (Fig. 5), we observed differences in both the total carbohydrate in the EPS samples and alterations in the sugar content (Table 5). As previously described [31], *Synechocystis* EPS is primarily composed of glucose (∼72%), fucose (∼10%), xylose (∼9%), mannose (∼5%), and galactose (∼3%) with trace percentages of ribose, rhamnose and N-acetyl glucosamine (Table 5). EPS samples from Δ*slr0977* showed a marked decrease in glucose composition (35%) compared to wild-type samples and a concomitant increase in the percentage of ribose (29%). One possibility is that in the absence of the native pore, increased activity of a *slr0977* gene product homolog leads to the improper transport of extracellular carbohydrates. In fact, this is likely due to the ABC transporter encoded by *sll0574-5*, as Δ(*slr0977*; Δ*sll0574-5*) demonstrated carbohydrate composition nearly identical to wild-type (Table 5). EPS derived from Δ*slr0982* and Δ*slr1610* showed a modest increase in the percentage of ribose from wild-type. While the EPS from the Δ*slr1610* showed a marked decrease in glucose composition (50%), the EPS of the Δ*slr0982* mutant was composed of 80% glucose. In addition, the EPS extracted from Δ*slr0982* contained less fucose than the wild-type. This may indicate that in the absence of *slr0982*, the *slr0977* gene product is capable of utilizing alternative ABC transporter components with a similar affinity for glucose as the *slr0982* gene product.

**Table 5:** Gas chromatographic analysis of EPS components^a^.

Sugar (Mol %)	Wild-type	Δslr0977	Δslr0982	Δslr1610	Δ(*slr0977*; Δ*sll0574-5*)	Δsll1213
glucose	71.7	35	80.8	50.4	73.8	76.3
fucose	9.7	10.1	1.1	10	9.3	-
xylose	8.6	6.9	0.3	8.7	7.1	2.8
mannose	5	3.4	6.9	3.9	4.5	9.8
galactose	3.1	9.9	6.3	9.5	3.2	7.4
ribose	0.6	29.4	4.1	15.3	1.2	2.8
rhamnose	0.6	5.4	–	2.2	0.3	–
N-acety glucosamine	0.6	–	0.5	–	0.7	1

a Composition of OAg from the indicated stain. Data are presented as the Mol% of each sugar from the total carbohydrate extracted. Sums may not add to 100% due to rounding.

It is possible that changes in EPS or OAg can lead to altered outer membrane protein (OMP) profiles, which may play a role in cellular attachment. To determine if this was the case with our mutants, we isolated OMPs from mutant strains and analyzed them by SDS-PAGE. Proteins were visualized by staining with Coomassie brilliant blue (Fig. 5B). This analysis demonstrated that the Δ*slr0982* strain lacked a protein with A molecular wieght of ∼250 kDa, which likely represents the monomer that comprises the proteinaceous S-layer. This result indicates an important role in fucose production and transport in the attachment of the S-layer to the cell surface, as the fucose minus strain, Δ*sll1213,* is also deficient in S-layer attachment [41].

As expected, no detectable fucose was present in the Δ*sll1213* strain, which is aberrant in cell wall structure, thylakoid membrane biogenesis, EPS as well as LPS production ([41] and Fig. 4). Aggregation of the Δ*sll1213* strain is detectable microscopically [41]. However, a defect in fucose production does not illicit autoflocculation from the culture medium. This may be due to the pleotropic effects of the Δ*sll1213* mutation, which compensate cell surface charge or structures by an unknown mechanism. However, the differences between these strains may also indicate that perturbations of the transport machinery, rather than the EPS or LPS content per se are responsible for the cellular aggregation.

Here we have bioinformatically identified over five hundred gene products associated cell surface structures in *Synechocystis* with homology to genes in *E. coli* and *Salmonella* (Table S1). These data included *slr0977*/*slr0982* and *slr0574-5* which were annotated as gene products in involved in OAg transport. We provide evidence that they are, in fact, involved in the transport of a similar carbohydrate structure, EPS. By modulating such cell surface moieties, strains can be developed for with autoflocculant phenotypes useful in biomass recovery. Additionally, genetically engineering cyanobacteria with altered cell surface carbohydrate structures in cyanobacteria will prove to be useful tools for bioremediation.

## Supporting Information

Table S1 Gene products potentially involved in the synthesis and assembly of the *Synechocystis* cell wall and cell-surface macromolecular components.
(XLS)

## References

[1] Liu X, Curtiss R 3rd (2009) Nickel-inducible lysis system in Synechocystis sp. PCC 6803. Proc Natl Acad Sci U S A 106: 21550–21554. 19995962 10.1073/pnas.0911953106 PMC2799798

[2] Liu X, Curtiss R 3rd (2012) Thermorecovery of cyanobacterial fatty acids at elevated temperatures. J Biotechnol 161: 445–449. 22944207 10.1016/j.jbiotec.2012.08.013

[3] Liu X, Sheng J, Curtiss R 3rd (2011) Fatty acid production in genetically modified cyanobacteria. Proc Natl Acad Sci U S A 108: 6899–6904. 21482809 10.1073/pnas.1103014108 PMC3084101

[4] Liu X, Fallon S, Sheng J, Curtiss R 3rd (2011) CO2-limitation-inducible Green Recovery of fatty acids from cyanobacterial biomass. Proc Natl Acad Sci U S A 108: 6905–6908. 21482802 10.1073/pnas.1103016108 PMC3084069

[5] Uduman N, Qi Y, Danquah MK, Forde GM, Hoadley A (2010) Dewatering of microalgal cultures: A major bottleneck to algae-based fuels. J Renew Sustain Ener 2: 012701.

[6] Pereira S, Micheletti E, Zille A, Santos A, Moradas-Ferreira P, et al (2011) Using extracellular polymeric substances (EPS)-producing cyanobacteria for the bioremediation of heavy metals: do cations compete for the EPS functional groups and also accumulate inside the cell? Microbiology 157: 451–458. 20966085 10.1099/mic.0.041038-0

[7] Micheletti E, Pereira S, Mannelli F, Moradas-Ferreira P, Tamagnini P, et al (2008) Sheathless mutant of Cyanobacterium Gloeothece sp. strain PCC 6909 with increased capacity to remove copper ions from aqueous solutions. Appl Environ Microbiol 74: 2797–2804. 18326679 10.1128/AEM.02212-07 PMC2394890

[8] Jittawuttipoka T, Planchon M, Spalla O, Benzerara K, Guyot F, et al (2013) Multidisciplinary evidences that Synechocystis PCC6803 exopolysaccharides operate in cell sedimentation and protection against salt and metal stresses. PLoS One 8: e55564. 23405172 10.1371/journal.pone.0055564 PMC3566033

[9] Sutherland I (2001) Biofilm exopolysaccharides: a strong and sticky framework. Microbiology 147: 3–9. 11160795 10.1099/00221287-147-1-3

[10] Flemming HC, Neu TR, Wozniak DJ (2007) The EPS matrix: the “house of biofilm cells”. J Bacteriol 189: 7945–7947. 17675377 10.1128/JB.00858-07 PMC2168682

[11] Davey ME, O’Toole G A (2000) Microbial biofilms: from ecology to molecular genetics. Microbiol Mol Biol Rev 64: 847–867. 11104821 10.1128/mmbr.64.4.847-867.2000 PMC99016

[12] Xu X, Khudyakov I, Wolk CP (1997) Lipopolysaccharide dependence of cyanophage sensitivity and aerobic nitrogen fixation in Anabaena sp. strain PCC 7120. J Bacteriol 179: 2884–2891. 9139904 10.1128/jb.179.9.2884-2891.1997 PMC179050

[13] Santander J, Robeson J (2007) Phage-resistance of Salmonella enterica serovar Enteritidis and pathogenesis in Caenorhabditis elegans is mediated by the lipopolysaccharide. Electron J Biotechn 10.

[14] Nikaido H (2003) Molecular basis of bacterial outer membrane permeability revisited. Microbiol Mol Biol Rev 67: 593–656. 14665678 10.1128/MMBR.67.4.593-656.2003 PMC309051

[15] Chen LZ, Wang GH, Hong S, Liu A, Li C, et al (2009) UV-B-induced oxidative damage and protective role of exopolysaccharides in desert cyanobacterium Microcoleus vaginatus. J Integr Plant Biol 51: 194–200. 19200158 10.1111/j.1744-7909.2008.00784.x

[16] Ozturk S, Aslim B, Suludere Z (2009) Evaluation of chromium(VI) removal behaviour by two isolates of Synechocystis sp. in terms of exopolysaccharide (EPS) production and monomer composition. Bioresour Technol 100: 5588–5593. 19560345 10.1016/j.biortech.2009.06.001

[17] Ozturk S, Aslim B, Suludere Z (2010) Cadmium(II) sequestration characteristics by two isolates of Synechocystis sp. in terms of exopolysaccharide (EPS) production and monomer composition. Bioresour Technol 101: 9742–9748. 20719501 10.1016/j.biortech.2010.07.105

[18] Ozturk S, Aslim B (2010) Modification of exopolysaccharide composition and production by three cyanobacterial isolates under salt stress. Environ Sci Pollut Res Int 17: 595–602. 19727881 10.1007/s11356-009-0233-2

[19] Lan SB, Wu L, Zhang DL, Hu CX, Liu YD (2010) Effects of drought and salt stresses on man-made cyanobacterial crusts. Eur J Soil Biol 46: 381–386.

[20] Samuel G, Reeves P (2003) Biosynthesis of O-antigens: genes and pathways involved in nucleotide sugar precursor synthesis and O-antigen assembly. Carbohydr Res 338: 2503–2519. 14670712 10.1016/j.carres.2003.07.009

[21] Kido N, Torgov VI, Sugiyama T, Uchiya K, Sugihara H, et al (1995) Expression of the O9 polysaccharide of Escherichia coli: sequencing of the E. coli O9 rfb gene cluster, characterization of mannosyl transferases, and evidence for an ATP-binding cassette transport system. J Bacteriol 177: 2178–2187. 7536735 10.1128/jb.177.8.2178-2187.1995 PMC176863

[22] Kaneko T, Tabata S (1997) Complete genome structure of the unicellular cyanobacterium Synechocystis sp. PCC6803. Plant Cell Physiol 38: 1171–1176. 9435137 10.1093/oxfordjournals.pcp.a029103

[23] Nakao M, Okamoto S, Kohara M, Fujishiro T, Fujisawa T, et al (2010) CyanoBase: the cyanobacteria genome database update 2010. Nucleic Acids Res 38: D379–381. 19880388 10.1093/nar/gkp915 PMC2808859

[24] Tatusov RL, Galperin MY, Natale DA, Koonin EV (2000) The COG database: a tool for genome-scale analysis of protein functions and evolution. Nucleic Acids Res 28: 33–36. 10592175 10.1093/nar/28.1.33 PMC102395

[25] Allen MM, Stanier RY (1968) Selective Isolation of Blue-green Algae from Water and Soil. Microbiology 51: 203–209. 10.1099/00221287-51-2-203 5652096

[26] Sambrook J, Fritsch EF, Maniatis T (1989) Molecular cloning: a laboratory manual. Plainview, NY: Cold Spring Harbor Laboratory Press.

[27] Ho SN, Hunt HD, Horton RM, Pullen JK, Pease LR (1989) Site-Directed Mutagenesis by Overlap Extension Using the Polymerase Chain-Reaction. Gene 77: 51–59. 2744487 10.1016/0378-1119(89)90358-2

[28] Ikeuchi M, Eggers B, Shen GZ, Webber A, Yu JJ, et al (1991) Cloning of the psbK gene from Synechocystis sp. PCC 6803 and characterization of photosystem II in mutants lacking PSII-K. J Biol Chem 266: 11111–11115. 1904061

[29] Yi EC, Hackett M (2000) Rapid isolation method for lipopolysaccharide and lipid A from gram-negative bacteria. Analyst 125: 651–656. 10892021 10.1039/b000368i

[30] Tsai CM, Frasch CE (1982) A sensitive silver stain for detecting lipopolysaccharides in polyacrylamide gels. Anal Biochem 119: 115–119. 6176137 10.1016/0003-2697(82)90673-x

[31] Panoff J-M, Priem B, Morvan H, Joset F (1988) Sulphated exopolysaccharides produced by two unicellular strains of cyanobacteria, *Synechocystis* PCC 6803 and 6714. Arch Microbiol 150: 558–563.

[32] Carlone GM, Thomas ML, Rumschlag HS, Sottnek FO (1986) Rapid microprocedure for isolating detergent-insoluble outer membrane proteins from Haemophilus species. J Clin Microbiol 24: 330–332. 3489731 10.1128/jcm.24.3.330-332.1986 PMC268907

[33] Merkle RK, Poppe I (1994) [1] Carbohydrate composition analysis of glycoconjugates by gas-liquid chromatography/mass spectrometry. In: William J. Lennarz GWH, editor. Methods Enzymol: Academic Press. 1–15. 10.1016/0076-6879(94)30003-8 8139491

[34] York WS, DAG, McNeil M, Stevenson TT, Albersheim P (1985) Isolation and characterization of plant cell walls and cell-wall components. *Methods Enzymol* 118: 3–40.

[35] Reeves PR, Hobbs M, Valvano MA, Skurnik M, Whitfield C, et al (1996) Bacterial polysaccharide synthesis and gene nomenclature. Trends Microbiol 4: 495–503. 9004408 10.1016/s0966-842x(97)82912-5

[36] Hausman BS, Williamson JA, Schreiner RP, Pulakat L, Gavini N (1998) The rfb genes in Azotobacter vinelandii are arranged in a rfbFGC gene cluster: a significant deviation to the arrangement of the rfb genes in Enterobacteriaceae. Biochem Biophys Res Commun 245: 572–582. 9571197 10.1006/bbrc.1998.8423

[37] Cuthbertson L, Powers J, Whitfield C (2005) The C-terminal domain of the nucleotide-binding domain protein Wzt determines substrate specificity in the ATP-binding cassette transporter for the lipopolysaccharide O-antigens in Escherichia coli serotypes O8 and O9a. J Biol Chem 280: 30310–30319. 15980069 10.1074/jbc.M504371200

[38] Blomfield IC, Vaughn V, Rest RF, Eisenstein BI (1991) Allelic Exchange in Escherichia-Coli Using the Bacillus-Subtilis Sacb Gene and a Temperature-Sensitive Psc101 Replicon. Mol Microbiol 5: 1447–1457. 1686293 10.1111/j.1365-2958.1991.tb00791.x

[39] Cuthbertson L, Kos V, Whitfield C (2010) ABC transporters involved in export of cell surface glycoconjugates. Microbiol Mol Biol Rev 74: 341–362. 20805402 10.1128/MMBR.00009-10 PMC2937517

[40] O’Toole G, Kaplan HB, Kolter R (2000) Biofilm formation as microbial development. Annu Rev Microbiol 54: 49–79. 11018124 10.1146/annurev.micro.54.1.49

[41] Mohamed HE, van de Meene AM, Roberson RW, Vermaas WF (2005) Myxoxanthophyll is required for normal cell wall structure and thylakoid organization in the cyanobacterium Synechocystis sp. strain PCC 6803. J Bacteriol 187: 6883–6892. 16199557 10.1128/JB.187.20.6883-6892.2005 PMC1251633

[42] Schmidt W, Drews G, Weckesser J, Mayer H (1980) Lipopolysaccharides in four strains of the unicellular cyanobacterium Synechocystis. Arch Microbiol 127: 217–222.

